# Unlocking the Full Potential of Health Care Teams: How Artificial Intelligence Can Help

**DOI:** 10.2196/77393

**Published:** 2026-05-11

**Authors:** Manchi (Monica) Hsu, Benny Bikash Pokharel, Jacqueline Kueper, Michaela Kerrissey, Sian Hsiang-Te Tsuei

**Affiliations:** 1 Faculty of Medicine, University of British Columbia, 317-2194 Health Sciences Mall, Vancouver, BC, V6T 1Z3, Canada, 1 6048229836; 2 Digital Trials Center, Scripps Research Translational Institute, La Jolla, CA, United States; 3 Department of Health Policy and Management, Harvard T.H. Chan School of Public Health, Harvard University, Boston, MA, United States; 4 Faculty of Health Sciences, Simon Fraser University, Burnaby, BC, Canada; 5 Department of Family Practice, Faculty of Medicine, University of British Columbia, Vancouver, BC, Canada

**Keywords:** team-based care, large language model, electronic medical records, artificial intelligence, AI, machine learning

## Abstract

Developing effective health care teams is critical to meet the rising complexity in patient care. However, optimizing team composition, interpersonal dynamics, and care processes in complex health care systems requires processing vast amounts of data that capture fluid interactions among professionals—a task that has been cumbersome, costly, and avoided by most organizations. Well-designed artificial intelligence (AI) tools can meaningfully advance the frontier of health care teamwork, but the application of AI in this regard has been lagging. To support this development, we outline the potential for AI to help optimize team composition, strengthen norms and relationships among professionals, and standardize team-based clinical care processes. These applications can improve the integration of health care teams. Given the importance of relevant data for realizing such advances, we describe the potential types and sources of data that can support AI development. Furthermore, we highlight enabling strategies, including data-sharing alliances and leadership engagement to address privacy, interoperability, and ethical considerations. We propose a sequenced roadmap for piloting these applications based on technological readiness and clinical feasibility, ensuring that human oversight remains central as AI tools are introduced into complex care environments.

## Introduction

Amid growing patient complexity, health professionals must increasingly rely on one another to deliver integrated, high-quality care [1]. Patients with multiple chronic illnesses often require care from a network of specialized providers—and more than 50% of the Canadian population aged 65 years and older have multimorbidity [2]. They may visit as many as 16 different clinicians in a single year, spanning multiple roles and care venues [3]. At the same time, a typical primary care physician will interact annually with 229 other physicians across 117 practices while managing 1200 to 1900 patients [4]. This presents a daunting coordination environment, and because of its complexity, teamwork has been vexingly difficult for health care organizations to efficiently and effectively improve.

The very complexity and distributed interdependence that make teams attractive for health care environments also make them difficult to define and implement in practice. However, organizational leaders often lack the time and cognitive capacity to optimize the design and operation of such teams because the team composition and tasks are often fluid and complex.

Artificial intelligence (AI) techniques and tools can help improve the design and operations of health care teams. Throughout this paper, we use “AI” as an umbrella term referring to a set of technologies including machine learning and deep learning, artificial neural networks, natural language processing, generative AI including large language models, and agentic AI systems. We exclude rule-based expert systems, physical robots, and robotic process automation [5]. The methods we consider in scope are included in 2 papers cited here [6,7] for readers who are interested.

Although AI tools have been introduced in many clinical areas [8], their application to arranging and organizing labor in health care remains largely untapped. Previous literature has largely focused on 2 separate domains. The work on teamwork in health care has examined the importance of organizational structures, collaboration, norms, and leadership styles without systematically drawing on AI tools [9-12]. On the flipside, AI development has largely focused on tools that directly influence administrative, academic, and clinical tasks, without directly influencing the team structure, dynamics, and operations [13-15]. At the intersection of these 2 topics lies a novel field of research that is needed to explore the role of AI in improving teamwork in health care.

Given the myriad ways AI may help team development, we drew on empirical and theoretical literature on care integration to focus on three key AI applications to health care teams: (1) optimizing team composition, (2) strengthening norms and relationships among professionals, and (3) standardizing team-based clinical care processes [1,16]. To illustrate the feasibility, we describe data sources (Table 1) and target tasks for AI techniques to unlock these 3 applications. We also suggest that data-sharing alliances—perhaps strengthened with federated learning or horizontal integration programs—may facilitate access to the necessary data for training AI models. When such applications become realized, ensuring that humans maintain the autonomy to interpret and implement the AI recommendations is also ethical and practical.

**Table 1.** Overview of data sources for artificial intelligence–driven team optimization.

Steps (definition) and dimensions captured		Data sources and examples
Application 1: identify optimal team members to care for target patient population		
Patient needs		EMR^a^: demographics, comorbidities, visit records, geographic data
Provider characteristics		Employment registry: provider demographics, training history, caseload records
Teamwork effectiveness		EMR: patient outcomes Validated survey of team performance^b^ [17]
Care outcomes		EMR: lab results, dates of patient-provider interactions, treatment records
Application 2: integrate new team members to cultivate effective organizational culture		
Provider preferences		EMR and voice recordings: provider-patient and provider-provider interactions. Validated surveys of personalities and preferences^b^ [18-20]
Organizational culture		Staff survey^b^^c^ [21]
Application 3: standardize clinical decisions to improve care quality and efficiency		
Clinical status		EMR: date, time, and results from patient-provider conversations, physical exams, lab tests, and imaging
Clinical decisions		EMR: treatment orders, care protocols
Patient-generated health data		Wearable devices, home monitoring devices, mobile tracking apps

a EMR: electronic medical record.

b The surveys are listed in Multimedia Appendix 1 [17-21] and have demonstrated good psychometric properties.

c Most surveys target the respondents’ view of the organizations. Wording may need to be adjusted to capture individual respondent’s views of themselves, which can then be analyzed to understand the respondents’ fit with each other.

## Application 1: Optimizing Team Composition

Because a patient’s care is often spread across units and shifts over time [11], observing and measuring the relationship between team composition and performance can be challenging. Without clearly defining such relationships, promptly optimizing team composition becomes problematic, which limits health care quality [22].

Training AI models to provide timely recommendations of appropriate members for a given patient’s care team requires learning relationships between the clinical environment, patient needs, and indicators of successful team performance. Not only can such research identify latent patterns driving referrals, but also researchers are directly piloting when multidisciplinary, proactive electronic consultations can be helpful. In 2 instances, proactive AI-driven predictions regarding the need for palliative care consultations improved access to palliative care while reducing subsequent hospitalizations [23,24]. Furthermore, a large quantity of relevant data is already available. For example, input variables may include existing and potential new provider profiles and patient clinical history including demographics, morbidities, and past experiences with providers from electronic medical records (EMRs). Outcome variables may include care performance based on EMR-derived metrics and team functions from validated surveys [17,25-27]. Users and experts can further refine the output based on disease-specific guidelines.

Within an episode of care, updated patient information in EMRs can dynamically adjust their optimal care team structure, potentially automating the consult process for relevant specialties. For instance, a patient admitted for pneumonia may initially only require a general internist, but if blood pressure drops precipitously, the AI tool may send out automatic alerts to engage an intensivist with tailored patient summaries that streamline the handoff process. Such AI tools may even discern the best person within a specialty to involve based on previous team-based interactions and care quality outcomes [26,27].

Beyond supporting individual patient care team assignment, patient panel data in EMRs can project health care needs [28-31], which can inform proactive workforce planning [26,27].

## Application 2: Strengthening Norms and Relationships Among Professionals

As health care teams expand to address varied and complex patient needs, aligning providers’ values and communication norms is essential for effective coordination and decision-making [1]. However, meaningfully, promptly, and systematically acculturating new members can be challenging given the financial and cognitive barriers facing purely human-led efforts.

AI tools can build on existing approaches to help identify the underlying organizational culture, team climate, and individual behavior within teams [12,32,33]. Such work has historically relied on time-consuming manual qualitative coding and quantification or numeric scale survey instruments that can be biased and context inappropriate. EMRs and recordings of providers’ interactions with patients and providers can reveal a team’s underlying attitude and preferences, in addition to past safety events and interpersonal conflicts [34,35]. Validated survey instruments can also supplement the identification of underlying personalities and preferences. Furthermore, AI tools can efficiently generate summarized versions of these organizational culture and climate factors for teams to consider potential improvements [36,37]. Further still, such discussions can be enhanced using currently available AI tools that introduce new members to effective communication skills [38,39]. Recent empirical studies on TeamVision, an AI-enhanced multimodal analytics platform, piloted the idea of capturing team dynamics using AI-powered tools into data-informed debriefs that can improve team cohesion and communication. This platform captures voice presence, automated transcripts, spatial orientation, and team interactions during health care simulations to identify communication patterns in real time. This was deployed across 56 teams (221 students) in a nursing curriculum. Both learners and educators rated the system as useful and trustworthy for strengthening relational dynamics [40].

AI-based onboarding is also generating momentum, although not yet widely applied [41]. AI tools can help analyze new members’ personalities and preferences based on surveys and previous patient interactions. Customized simulation exercises can then help new members align with the team [42].

## Application 3: Standardizing Clinical Care Processes

Despite clinical guidelines’ supposed benefits, clinicians may voluntarily or inadvertently reject guideline recommendations [43,44]. These situations can lead to confusion and problematic variation across care teams where individual clinicians exhibit highly varying practice patterns. Standardizing clinical processes on demand can set a floor for care quality and efficiency, but its effectiveness is limited by the constant human oversight required to implement them well.

EMRs already help to standardize clinical workflow via clinical decision support systems that codify clinical decisions, allowing different care providers to understand and continue subsequent care delivery. Furthermore, many AI tools already use EMR and multimodal data for pattern identification and risk predictions, ranging from disease progression and clinical care complications to treatment outcomes [45-51]. EMR data can capture patient needs in demographics, diagnoses, tests, and images; clinical interventions in drugs, procedures, and psychotherapies; and clinical outcomes in tests and images. Even more up-to-date insights into a patient’s health status can leverage patient-generated data from wearable devices, home monitoring devices, and mobile tracking apps [52-61].

AI tools may recommend preventative actions, disease management courses, and diagnostic recommendations based on up-to-date patient information and clinical guidelines. If multiple providers can consistently access the AI recommendations, care standardization may improve alongside better care coordination and minimize information loss.

Recent large-scale empirical studies demonstrate that AI-assisted decision support can meaningfully improve adherence to evidence-based care while reducing variability and diagnostic errors. In a prospective study spanning 39,849 primary care visits across 15 clinics in Nairobi, an AI consult tool provided real-time background diagnostic support. Clinicians using the tool experienced substantial error reductions in taking history (32%), ordering investigations (10%), diagnosing (16%), and treating (13%), and improvements persisted throughout the study period [62].

## Implications

In what follows, we discuss the practical and ethical implications of applying AI to teamwork in health care. We start with the need for relevant data, highlighting the importance of and policy supports for data-sharing alliances. We then describe how the data need to be adjusted to achieve optimal results and end with a discussion of ethical implications.

## Practical Need for Data-Sharing Alliance

Large datasets that capture sufficient variability will be key for AI tools to learn reliable and useful patterns. Smaller health care organizations with limited information management capabilities, little patient variability, and few employees may have difficulty generating such datasets independently. Three solutions may encourage the development of data-sharing alliances in the current landscape where data are prized as a valuable resource.

First, federated learning enables health care organizations to collaborate on AI model development by maintaining control over their own data without pooling sensitive patient data into a single location, thereby addressing data privacy concerns. Each organization keeps its data securely on-site while sharing only model updates, so the combined system learns from all participants [63]. For instance, this approach is used by the Mayo Clinic Platform Connect and has proven to be successful in generating a prediction model forecasting patient responses to chemotherapy [64].

Second, accountable care organization policy encourages integration across health care organizations. Financial subsidies can incentivize uniform infrastructure standards or regulatory safe harbors for innovation pilots [65]. For example, they can also drive data sharing by adopting interoperability standards like Fast Healthcare Interoperability Resources (FHIR), which outlines a set of data formats and elements for digital health data transfer,

Third, strengthening health care organization leaders’ trust, appreciation, and context-appropriate adaptation of AI tools’ capacity for informing teamwork will be crucial. Executive champions can set organizational priorities, allocate resources, and communicate a clear vision for AI adoption. Structured change management strategies—including clinician training on digital literacy, iterative feedback loops, and transparent communication—help build trust and facilitate culture change across clinical teams. Finally, multidisciplinary governance committees can integrate ethical oversight, technical standards, and clinical priorities into operational and clinical decision-making, ensuring that AI implementation remains accountable, safe, and sustainable over time.

## Practical Nuances for Data Considerations

For AI tools to provide up-to-date recommendations on team compositions, the data need to reflect day-to-day changes in patient acuity and clinician availability. AI developers will need to optimize how frequently to update the data to best reflect real-time clinical needs and clinician resources (eg, caseload and emergent expertise) without driving alert fatigue or administrative burnout.

Given the potential for AI tools to consider diverse types of variables, ensuring that multiple outcomes are assessed and incorporated into the models can be helpful. For example, AI developers can parameterize care continuity to minimize care disruptions alongside other variables that may affect care quality.

## Practical Considerations on Adoption Resistance

Integrating new technology into existing workflows can face considerable resistance [66]. The range of factors can include—but are not limited to—concern over loss of power, past experiences of novel technologies, and general resistance to change. Integration of AI tools into teamwork in health care can reveal similar challenges [67] and may especially present concerns about surveillance and privacy in work interactions. These challenges require thoughtful AI implementation strategies that take team members’ concerns seriously and seek to address them. This can include transparent communication from leadership, engagement of frontline voices in tool development and testing, and responsive governance structures. These efforts may be enhanced by (1) focusing on common values, (2) fostering collaborative vision, (3) garnering buy-in around the technology, and (4) supporting psychological safety [68,69]. Targeted training, incentives, and ongoing workflow adjustments would be key to supporting additional uptake [68]. Finally, given the complex ethical and legal risks around AI, providing a well-developed legal and ethical framework would also minimize associated anxiety regarding AI use [68].

## Ethical Considerations Around Human Oversight and Insight

The applications outlined previously show that AI can inform team-based care, but ultimately, teamwork is a human process. This has both ethical and practical implications [70].

First, because AI tools used in team-based care directly shape human work experiences, health professionals must retain the autonomy to accept, refine, or reject AI-generated recommendations. However, this autonomy may be problematic due to the phenomenon of automation bias—the tendency to overrely on algorithmic outputs even when flawed. Users may mechanically adhere to AI recommendations without critical engagement [71]. Helping health care providers develop the relevant skills to safely use AI for clinical work may require time and investment.

Second, AI systems trained on historical data risk propagating structural biases, particularly if marginalized groups are underrepresented in training datasets [72]. Without careful development, evaluation, and oversight, these biases could influence team composition, decision-making hierarchies, and ultimately patient outcomes, reinforcing existing power asymmetries within health care [73]. Explicit attention to this potential for bias requires reviewing and critically assessing model output for bias potential.

Third, questions of responsibility and liability arise when AI recommendations shape team-based decisions, but final accountability remains with clinicians [74]. Clear governance structures and continuous evaluation processes are therefore essential to delineate professional responsibility, ensure transparency, and maintain trust.

Fourth, the impact on professional roles must be considered: while AI can standardize workflows and enhance efficiency, it could also shift authority from clinicians to algorithm developers or organizational leaders, reshaping professional boundaries in ways that demand scrutiny [75].

Finally, because teamwork involves interpretation, negotiation, and shared responsibility, AI-generated recommendations should be seen not as prescriptive directives but as tools to initiate dialogue—surfacing problems, questions, and options for deeper engagement rather than replacing human judgment.

## Conclusions

Despite efforts to enhance team-based care, humans are inherently limited by the quantity of data they can meaningfully experience and analyze. AI tools can leverage the considerable data that human interactions in complex health care systems generate to help optimize team composition, improve interpersonal interactions, and standardize care processes. Given the current lack of empirical data and systematic synthesis on this topic, this paper conceptually frames the discussion around leveraging AI tools to improve team-based care. This can motivate further inquiry, and we offer tangible recommendations about how such work can proceed, based on technological readiness.

The varying approaches offer different opportunities for a variety of groups. Policymakers can draw on evidence around privacy, ethics, and related concerns to develop legislation, regulations, guidelines, and incentives. Practitioners play a key role by engaging with policymakers and organizations to codevelop pilot initiatives and provide feedback on feasibility and impact. Meanwhile, AI developers can build into their tools interoperable elements while adhering to emerging policies.

Finally, sequencing these applications according to technological readiness and clinical need may be helpful. In the short term, standardizing clinical processes is immediately actionable, as AI-assisted order sets and decision support tools have already demonstrated significant value [76,77]. In the midterm, optimizing team compositions can be operationalized by leveraging existing datasets such as bed management dashboards and scheduling tools. Targeted pilots may provide early evidence of feasibility and impact. Strengthening norms and relationships may be a longer-term goal, given the ethical, cultural, and privacy challenges of collecting and analyzing interpersonal data within health care teams.

## Supplementary material

10.2196/77393 Multimedia Appendix 1 Validated surveys for artificial intelligence–driven team optimization.

## Abbreviations

AI: artificial intelligence
EMR: electronic medical record
FHIR: Fast Healthcare Interoperability Resources
