# Supplementary material for: Unlocking the Full Potential of Health Care Teams: How Artificial Intelligence Can Help
Source: JMIR AI. 2026 May 11;5:e77393. doi: 10.2196/77393 (PMC13160488; doi:10.2196/77393)
Supplement: Multimedia Appendix 1 [file ai-v5-e77393-s001.docx]

**Appendix A**

Table A1 describes the validated surveys that can be used for artificial intelligence-driven team optimization.

Table A1. Validated Surveys for Artificial Intelligence-Driven Team Optimization

| Dimension | Survey Name |
| --- | --- |
| Teamwork Effectiveness [1] | Cross-function Team Process Survey  Psychological Safety and Team Learning  Team Effectiveness Audit Tool  Team Process Survey  Team Survey  Team Effectiveness Survey  Teamwork Quality Survey  Team Emergency Assessment Measure  Relational Coordination Survey  Nursing Teamwork Survey |
| Provider Personality and Preference [2–4] | Neo-five factor Inventories  Attitude Toward Health Care Team Scale  TeamSTEPPS Teamwork Attitudes Questionnaire |
| Organizational Culture [5] | Survey of Organizational Culture  Corporate Culture Questionnaire  Organizational Culture Survey |

References

1. Valentine MA, Nembhard IM, Edmondson AC. Measuring Teamwork in Health Care Settings: A Review of Survey Instruments. Medical Care. 2015 Apr;53(4):e16.

2. Assessing teamwork attitudes in healthcare: development of the TeamSTEPPS teamwork attitudes questionnaire - PubMed [Internet]. [cited 2025 Sep 25]. Available from: https://pubmed.ncbi.nlm.nih.gov/20702444/

3. A new look at the big five factor structure through exploratory structural equation modeling - PubMed [Internet]. [cited 2025 Sep 25]. Available from: https://pubmed.ncbi.nlm.nih.gov/20822261/

4. Development of an Attitudes Toward Health Care Teams Scale - PubMed [Internet]. [cited 2025 Sep 25]. Available from: https://pubmed.ncbi.nlm.nih.gov/10350960/

5. Scott T, Mannion R, Davies H, Marshall M. The Quantitative Measurement of Organizational Culture in Health Care: A Review of the Available Instruments. Health Serv Res. 2003 Jun;38(3):923–45.
